# Supplementary material for: Content-rich biological network constructed by mining PubMed abstracts
Source: BMC Bioinformatics. 2004 Oct 8;5:147. doi: 10.1186/1471-2105-5-147 (PMC528731; doi:10.1186/1471-2105-5-147)
Supplement: Additional File 2 — The original results of the above study (non-essential files are deleted to keep the file size under the limit set by BMC bioinformatics). [file 1471-2105-5-147-S2.bz2 › chilibotAdditionalFile2/dip10/691ID3056932E18012/html/HISJ_HISQ.html]

 


 **HISJ** and **HISQ** 
  
Found 12 abstracts in PubMed, retrieved 10.  
 

 What does Google say? 
 PDF only 
| .edu only 

---

**Interactive relationship** (e.g. stimulation, inhibition, etc)

**Neutral relationship**- The reconstituted system is entirely dependent on both the periplasmic protein,  **HisJ** , and the membrane bound complex, composed of proteins  **HisQ** , HisM, and HisP.  Ref: 2647746 J Biol Chem, 1989
- They also suggest that the REP Repetitive Extragenic Palindromic sequences, which are located in the  **hisJ**   **hisQ**  intercistronic region, may interfere with translation of the  **hisQ**  gene and affect upstream messenger RNA stability by protecting it from 3 to 5 nuclease degradation in agreement with data presented by Newbury et al.  Ref: 3130541 Mol Microbiol, 1988

**Non-interactive relationship** (e.g. studied together, co-existance, homology, etc.)

- In this in vitro preparation, transport activity is completely dependent on the presence of all four permease proteins  **HisJ** ,  **HisQ** , HisM, and HisP and on internal ATP.  Ref: 2674940 Proc Natl Acad Sci U S A, 1989
- The reconstituted system depends on the presence of the membrane bound permease proteins,  **HisQ** , HisM, and HisP, and does not function if reconstitution is performed in the presence of a mutant  **HisJ**  protein, HisJ5625, that can bind histidine normally but can t interact properly with the membrane complex.  Ref: 2645283 J Biol Chem, 1989
- The nucleotide sequence of the entire histidine transport operon from Salmonella typhimurium has been determined and is shown to consist of four genes,  **hisJ** ,  **hisQ** , hisM and hisP.  Ref: 7050725 Nature, 1982
- The high affinity histidine permease of Salmonella typhimurium is encoded by a four gene operon containing a large intercistronic region located between the first gene  **hisJ**  and the three distal genes  **hisQ** , hisM, hisP.  Ref: 3130541 Mol Microbiol, 1988
- The bacterial histidine permease, an ABC transporter, from Salmonella typhimurium is composed of a membrane bound complex, HisQMP2, comprising two hydrophobic subunits  **HisQ**  and HisM, two copies of an ATP hydrolyzing subunit, HisP, and a soluble receptor,  **HisJ** .  Ref: 11456221 J Bioenerg Biomembr, 2001
